# Flexible Structure of Peptide-Bound Filamin A Mechanosensor Domain Pair 20–21

**DOI:** 10.1371/journal.pone.0136969

**Published:** 2015-08-31

**Authors:** Jonne Seppälä, Helena Tossavainen, Nebojsa Rodic, Perttu Permi, Ulla Pentikäinen, Jari Ylänne

**Affiliations:** 1 Department of Biological and Environmental Science and Nanoscience Center, University of Jyväskylä, Jyväskylä, Finland; 2 Program in Structural Biology and Biophysics, Institute of Biotechnology, University of Helsinki, Helsinki, Finland; Universitetet i Bergen, NORWAY

## Abstract

Filamins (FLNs) are large, multidomain actin cross-linking proteins with diverse functions. Besides regulating the actin cytoskeleton, they serve as important links between the extracellular matrix and the cytoskeleton by binding cell surface receptors, functioning as scaffolds for signaling proteins, and binding several other cytoskeletal proteins that regulate cell adhesion dynamics. Structurally, FLNs are formed of an amino terminal actin-binding domain followed by 24 immunoglobulin-like domains (IgFLNs). Recent studies have demonstrated that myosin-mediated contractile forces can reveal hidden protein binding sites in the domain pairs IgFLNa18**–**19 and 20**–**21, enabling FLNs to transduce mechanical signals in cells. The atomic structures of these mechanosensor domain pairs in the resting state are known, as well as the structures of individual IgFLN21 with ligand peptides. However, little experimental data is available on how interacting protein binding deforms the domain pair structures. Here, using small-angle x-ray scattering-based modelling, x-ray crystallography, and NMR, we show that the adaptor protein migfilin-derived peptide-bound structure of IgFLNa20**–**21 is flexible and adopts distinctive conformations depending on the presence or absence of the interacting peptide. The conformational changes reported here may be common for all peptides and may play a role in the mechanosensor function of the site.

## Introduction

Filamins (FLNs) are large, multi-domain rod-like proteins initially found to crosslink actin filaments that regulate the stability and viscoelastic properties of the actin cytoskeleton [1]. Since their discovery, knowledge about their cellular functions has been broadened by the discovery of a wide array of interacting partners with diverse functions. These include transmembrane receptors, intracellular signaling molecules, and cytoskeletal proteins. Thus, FLNs link the extracellular matrix to the cytoskeleton, function as a scaffold during signaling events, and regulate cell adhesion dynamics [2–4]. Recently, it has become evident that FLNs also detect local physical forces and play a role in the mechanosensing that helps cells to respond to mechanical cues [5–12].

**Fig 1:**
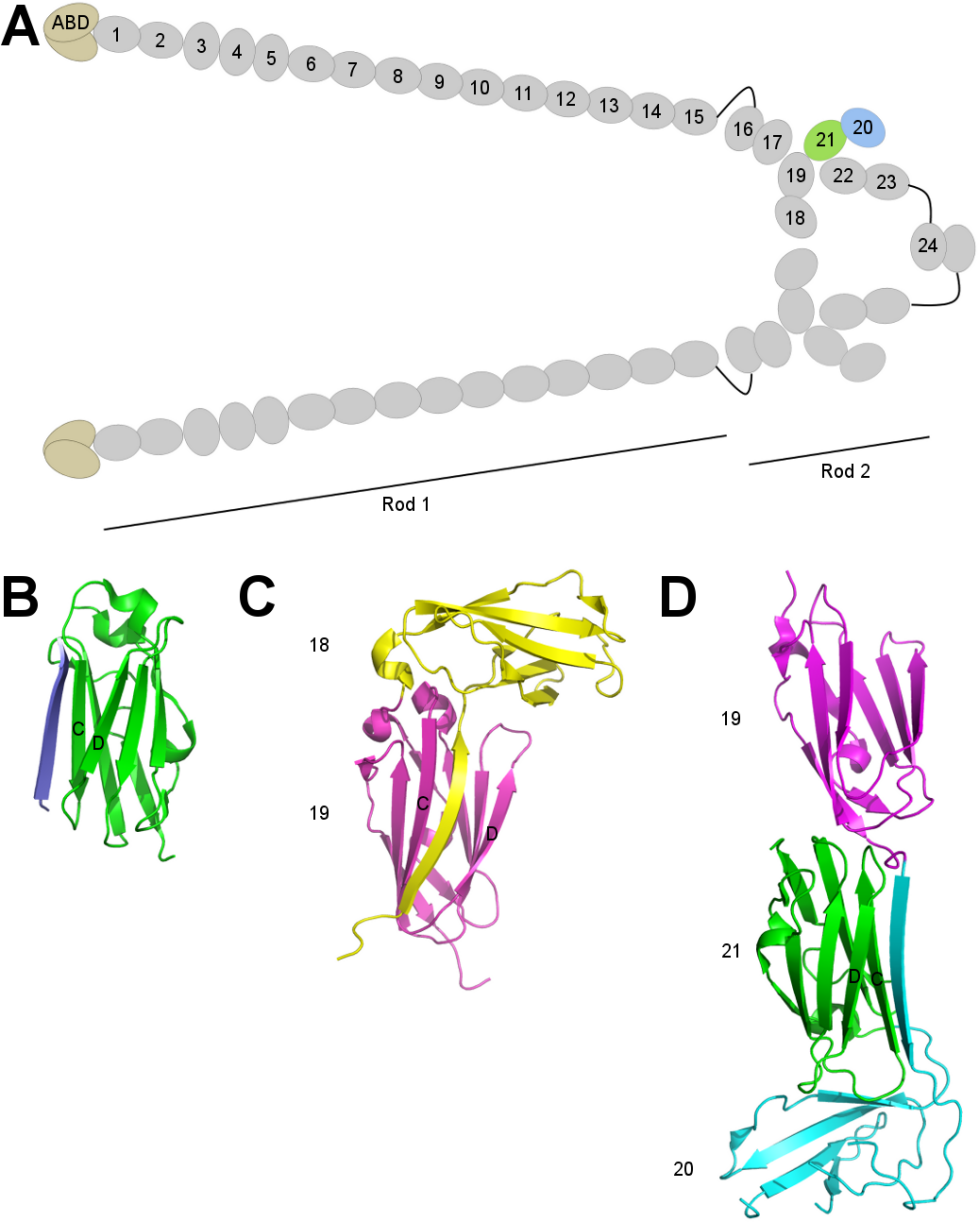
Structures of FLNs. **A** A schematic representation of FLN dimer. An N-terminal actin binding domain (ABD) is followed by 24 Ig-like repeats that are traditionally divided into two rods separated by a small flexible hinge in between. The most C-terminal repeat mediates the dimerization. The colored domains were studied here. **B** Ligands bind to IgFLNs via β sheet augmentation. Structure of migfilin peptide (blue) bound to the CD face of IgFLNa21 (green) (PDB ID:2W0P) [18]. **C–D**, Structures of FLN mechanosensor modules, IgFLNa18**–**19 (2K7Q) [24] and IgFLNa19**–**21 (2J3S) [23]. The A strands of IgFLNa18 (yellow) and 20 (cyan) bury the ligand binding interfaces of IgFLNa19 (magenta) and 21 (green).

In vertebrates, the FLN family comprises three highly conserved proteins: FLNa, FLNb, and FLNc. FLNa is the most abundant and widely expressed isoform along with FLNb, whereas the expression of FLNc is more restricted [13]. FLNs are composed of an N-terminal actin-binding domain followed by a string of 24 filamin immunoglobulin domains (IgFLNs), typically divided into rod 1 and 2 through two flexible hinge regions between domains 15**–**16 and 23**–**24, respectively (Fig 1A) [1,13]. The most C-terminal IgFLN mediates self-association, thus forming a dimer needed in the cross-linking of actin filaments [14]. The other IgFLNs function as interaction modules. The majority of the known interacting partners have been mapped to bind the rod 2 domains, whereas domains 9**–**15 of rod 1 have a secondary actin-binding site [2,4,15].

IgFLNs are β sandwiches in which seven β strands are assembled into two β sheets [13]. The face formed by the C and D strands forms a general ligand-binding interface [12,16]. Currently, in all known heteromolecular complex structures of IgFLNs, the peptide ligands interact with a mechanism called β sheet augmentation, in which the peptide forms an additional antiparallel β strand next to the C strand of IgFLNs (Fig 1B) [12,16–21]. The dimerization interface of IgFLN24 is mediated by β sheet augmentation through the β strand D [14,22].

Structural studies have shown that not all IgFLNs are arranged linearly as beads-on-a-string, but form multidomain modules whose functions are not always known (Fig 1A) [21,23–26]. IgFLNa18**–**19 and 20**–**21 have an exceptional inter-domain interaction in which the first β strand of the preceding domain folds along the ligand-binding face (i.e. the CD face) of the following domain, mimicking the β sheet-forming peptide ligands (Fig 1C and 1D). This arrangement has been shown to auto-inhibit ligand binding [23] and to provide a mechanism for mechanical regulation, where external forces can unfold the masking β strand and enable ligand binding [8,9,12]. The mechanical regulation of these mechanosensor domain pairs has been recently verified [10] and shown to take place within physiological force rates generated by myosin [27,28].

The ligand-binding to individual IgFLN domains has been extensively studied [12]. However, it is not known how the interacting protein binding deforms the structure of mechanosensor domain pairs. To address this question, we performed small-angle x-ray scattering (SAXS) studies for IgFLNa20**–**21 with a cell adhesion regulation-related adaptor protein migfilin [29] peptide. In addition, we solved the crystal structure of IgFLNa20–21 lacking the ligand-binding inhibitory β strand, called hereafter IgFLNaΔA20**–**21, in the complex with the peptide. The study revealed that the peptide-bound structure is flexible and adopts distinctive conformations from the unbound form or the one lacking the inhibitory β strand.

## Materials and Methods

### Recombinant proteins

The IgFLNa20–21 (residues 2141**–**2329 according to domain boundaries in [13]) and IgFLNa∆A20**–**21 (2151**–**2329) fragments were generated by polymerase chain reaction and cloned into a modified pGEX vector (GE Healthcare). The inserts were verified by sequencing. The glutathione *S*-transferase fusion proteins were expressed in *Escherichia coli* BL21 cells at 37°C for 4 h. The cells were lysed at 2000 PSI using a French Pressure Cell Press (Thermo Fisher Scientific). The lysates were centrifuged at 48000*g* for 30 min and subsequently purified with Glutathione Sepharose 4 Fast Flow (GE Healthcare) according to the manufacturer’s instructions. Glutathione *S*-transferase was cleaved with tobacco etch virus protease at 4°C for 16 h and removed from the solution with the Glutathione Sepharose. The proteins were further purified with size-exclusion chromatography in 20 mM Tris at pH 8.0, 100 mM NaCl, and 1 mM DTT using a Superdex 75 HR 26/60 column (GE Healthcare) on an Äkta Prime FPLC system (GE Healthcare), and finally concentrated using Amicon Ultra-15 (Millipore) filter units. The purity of the proteins was confirmed with SDS-PAGE and the monodispersity was verified with analytical gel filtration using a Superdex 75 HR 10/30 column (GE Healthcare).

### Small-angle x-ray scattering

SAXS data were collected at the European Synchrotron Radiation Facility (Grenoble, France), beamline BM29 [30] (S1 Table). The data were collected at 277 K in 20 mM Tris (pH 8.0), 100 mM NaCl, and 10 mM DTT using 1**–**4 mg/ml concentrations of IgFLNa20**–**21 and IgFLNa∆A20**–**21. Two times molar excess of the migfilin peptide was used to ensure saturation in binding. The small size of the peptide enabled direct subtraction of its scattering when applied also to the sample buffer. A PILATUS 1M image plate was used, at a sample/detector distance of 2.85 m and wavelength of 0.10 Å, covering the momentum transfer range of 0.01 < *q* < 5 nm^-1^ (*q* = 4ᴨsin(θ)/λ where 2θ is the scattering angle). The data were processed using the standard procedures of the ATSAS program package [31]. Buffer subtractions were conducted with PRIMUS [32]. The radius of gyration *R*_*g*_ was estimated with AUTORG [33] and distance distribution functions *P(r)* and particle maximum dimension *D*_*max*_ were estimated using DATGNOM [34]. DATPOROD was used to estimate the excluded volume (*V*_*p*_) of the hydrated particle [31]. Dimensionless Kratky (*qR*_*g*_ *2 x I(q)/I(0) versus qR*_*g*_ for *V*_*c*_ normalized and *qV*_*c*_ *2 x I(q)/I(0) versus qV*_*c*_ for volume-of-correlation *V*_*c*_ normalized) [35] and Porod–Debye (*q4 x I(q) versus q4*) [36] plots were used to assess the flexibility of the proteins. *V*_*c*_ was calculated with the program SCÅTTER [37]. The bead-modeling program DAMMIF [38] was used to generate an *ab initio* model of IgFLNa20**–**21. Ten individual runs of DAMMIF were performed and averaged with DAMAVER [39]. CRYSOL [40] was used to evaluate the scattering of the IgFLNa20–21 crystal structure (from IgFLNa19**–**21 crystal structure, PDB ID: 2J3S) [23]. SUPCOMB [41] was used to overlay the crystal structure and the *ab initio* model with minimal normalized spatial discrepancy. An ensemble optimization method (EOM) [42] was used to further model the inter-domain flexibility and size distribution in solution. First, a pool of 10,000 randomly generated models of IgFLNa20–21 and IgFLNa∆A20**–**21 were generated with the RanCh program. The inter-domain linker (residues 2230–2236) and IgFLNa20 A strand (residues 2141–2150) were considered to be random chains. Then, a genetic algorithm program, GAJOE, was used to select an ensemble of 20 models whose combined scattering best fit with the experimental scattering. The data along with the *ab initio* model were submitted to SASBDB [43].

### Crystallography

IgFLNa∆A20**–**21 in complex with migfilin peptide (^5^PEKRVASSVFITLAPPRR DVAVAE^28^, EZBiolab, Westfield, IN) was crystallized using the hanging drop vapor diffusion method at 295 K with an equimolar (1 mM) protein-peptide mixture. Next, 2 μl droplets containing equal volumes of the protein-peptide mixture and 0.1 M MES at pH 6, 1.9 M (NH_4_)_2_SO_4_, 0.1 M (CH_3_CO_2_)_3_Pr were equilibrated against 1 ml of the reservoir solution. The crystals were transferred to 25% glycerol in the reservoir solution before freezing under liquid nitrogen. The data were collected at 100 K at the European Synchrotron Radiation Facility (Grenoble, France), beamline ID14**–**1, using the ADSC Q210 CCD detector, and were processed using the XDS program package [44]. The crystal structure was solved by molecular replacement with Phaser [45] using the structure of IgFLNa21 (PDB code: 2W0P, A chain) [18] as a search model. The model was built using ARP/wARP [46] and Coot [47] and refined using REFMAC 5.5 [48]. TLS refinement parameters were defined with help of the TLSMD server [49]. Final refinement was made using the PDB-REDO server to optimize the refinement parameters [50]. Structural factors and atomic coordinates were deposited in the PDB with ID 4P3W. All crystallographic figures were generated with PyMOL (Schrödinger LCC, Portland, OR).

### NMR

Migfilin peptide binding was monitored by acquiring a ^1^H, ^15^N HSQC spectrum of samples with protein-to-peptide concentration ratios of 1:0.0, 1:0.5, 1:1, 1:3, and 1:5. Backbone chemical shift assignment was performed for free IgFLNΔAa20–21 and the 1:5 IgFLNaΔA20–21:migfilin complex with HNCACB and CBCA(CO)NH spectra, the latter having phase-inverted signals for Cβ of residues Ala, Ile, Val, Thr, and Cα of Gly. ^15^N T_1_ and T_2_ relaxation data were acquired with the following time points: 10, 60, 110, 330, 660, 920, 1200, 1500, 2100, and 2700 ms, and additionally 3500 ms for the complex (T_1_) and 10, 30, 50, 70, 90, and 110 ms (T_2_). An exponentially decaying curve was fitted to the peak intensities, as implemented in the program Sparky (T.D. Goddard and D.G. Kneller, SPARKY 3, University of California, San Francisco). To study amide protection, the protein sample in H_2_O was lyophilized and subsequently dissolved in D_2_O. The exchange was followed by a series of ^1^H, ^15^N HSQC spectra, extending to 23 h for the free form and to 63 h for the complex form. The time before the start of the first spectrum was approximately 18 min. All aforementioned spectra were recorded at 30°C on a Varian INOVA 800 MHz spectrometer equipped with a cryogenically cooled ^1^H, ^13^C, ^15^N z-gradient probehead. Diffusion data was recorded at 30°C on a Bruker AVANCE III HD 600 MHz spectrometer equipped with a ^1^H, ^13^C, ^15^N z-gradient cryoprobe. Gradient strength range was 2.4 to 47.2 G/cm. The data were analyzed with the built-in Dynamics Center program using intensities of manually picked peaks.

## Results

### Migfilin binding changes the conformation of IgFLNa20–21

To study how the interacting protein binding deforms the structure of the IgFLNa20**–**21 mechanosensor domain pair, we performed extensive SAXS measurements with a model peptide derived from migfilin. All measurements were done in three different protein concentrations and the Guinier analysis indicated no apparent particle aggregation or repulsion (S1 Fig). The SAXS-derived structural parameters of each concentration and the combined data are given in Table 1. The molecular weights calculated from the Porod volumes (*V*_*p*_) indicate monomeric species in both the absence and presence of migfilin peptide.

**Table 1:** SAXS derived structure parameters for IgFLNa20–21 and IgFLNa∆A20–21 with and without bound migfilin peptide.

Sample	*c* (mg/ml)	*R*_*g*_ (nm)^a^	*D*_*max*_ (nm)^b^	*V*_*p*_ (nm^3^)^c^	*Mw* (kDa)^d^
***IgFLNa20-21***	1	1.9	6.7	32.7	19.2
	2.5	2.0	7.0	32.0	18.8
	4	2.0	6.9	32.2	18.9
		**1.9**	**6.8**	**32.0**	**18.8**
***IgFLNa20-21+migfilin***	1	2.3	8.0	33.3	19.6
	2.5	2.3	8.1	34.3	20.2
	4	2.4	8.4	35.0	20.6
		**2.3**	**8.2**	**32.9**	**19.4**
***IgFLNa∆A20-21***	1	2.4	8.5	27.8	16.4
	2.5	2.4	8.5	28.1	16.5
	4	2.4	8.4	28.1	16.5
		**2.4**	**8.5**	**28.9**	**17.0**
***IgFLNa∆A20-21+migfilin***	1	2.4	8.5	31.0	18.2
	2.5	2.5	8.4	31.0	18.2
	4	2.5	8.6	30.6	18.0
		**2.4**	**8.2**	**31.1**	**18.3**

The values showed in bold are for the merged scattering data of high and low concentration for each sample. See related S2 Table for additional parameters.

^a^ From Guinier analysis.

^b^ Estimate from *P(r)* calculation in DATGNOM [34]

^c^ Estimated from the regularized scattering obtained from *P(r)* calculation in DATPOROD [31].

^d^ Estimated from the hydrated particle volume *V*_*p*_ by dividing the volume by 1.7 [31]. Calculated monomeric molecular weights from sequence are approximately 20.0 kDa for IgFLNa20**–**21, 18.7 kDa for IgFLNa∆A20**–**21, and 2.6 kDa for the migfilin^5–28^ peptide.

**Fig 2:**
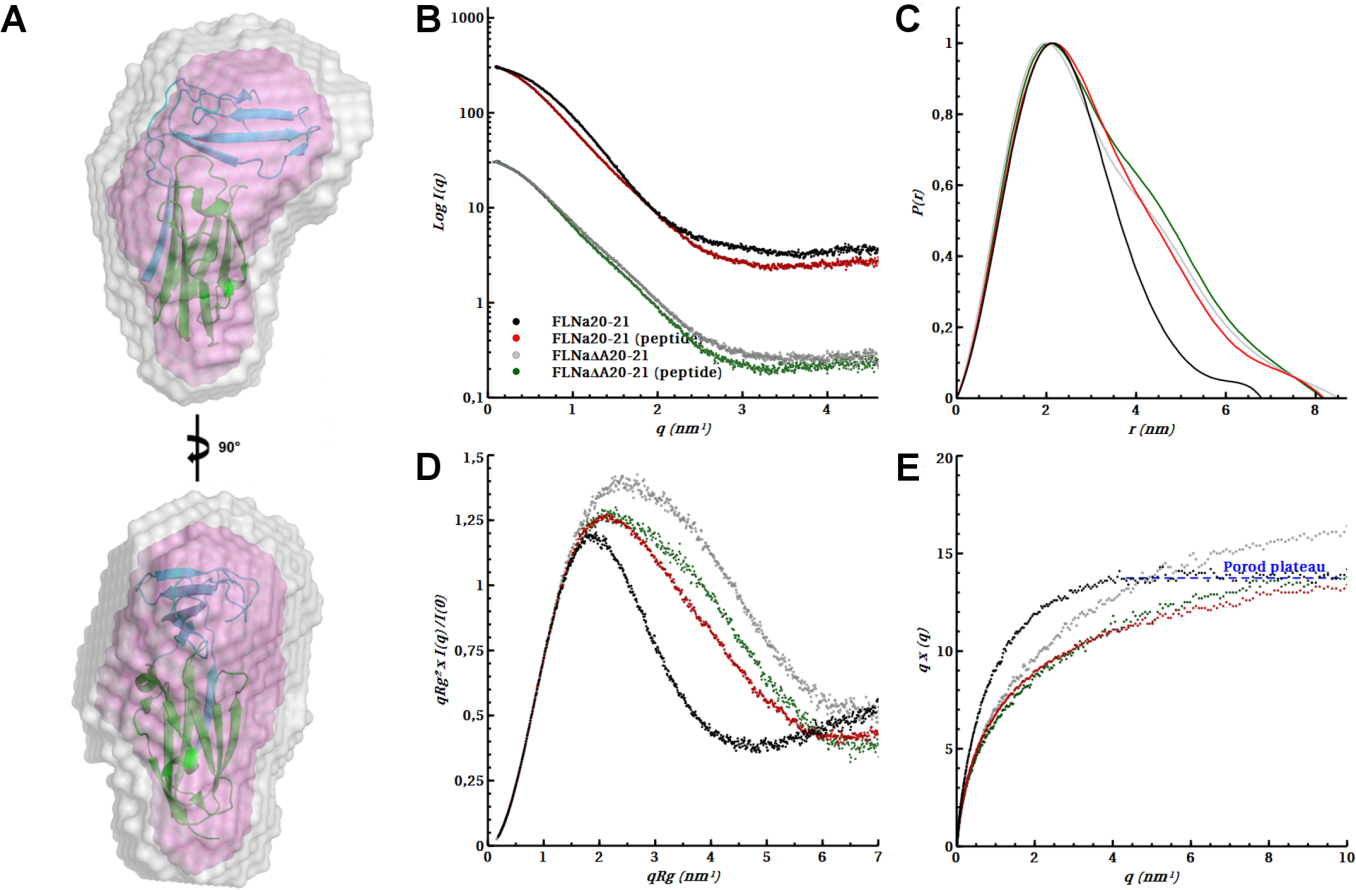
Standard analysis of SAXS data. **A** *Ab initio* modeling of IgFLNa20-21. The averaged *ab initio* model (in magenta) is overlaid with the envelope of all individual *ab initio* models superposed with minimal normalized spatial discrepancy (in gray). The crystal structure of IgFLNa20**–**21 (from 2J3S [23] is superposed to the averaged *ab initio* model. **B** Experimental scattering and Guinier fits (*inlet*), **C** Distance distribution function *P(r)*, **D** *R*_*g*_ normalized Kratky plots (for Vc normalized Kratky plots, see S3 Fig), and **E** Porod-Debye plots of IgFLNa20**–**21 and IgFLNa∆A20**–**21 with and without migfilin peptide. The data is shown for the merged scattering of high and low concentration data scaled to same forward scattering intensity *I(0)* except for Guinier plots which are arbitrarily displaced on the y axis for clarity.

The analysis of the SAXS data showed that IgFLNa20**–**21 is a compact structure in solution. The shape of the distance distribution function *P(r)* is a bell-shaped curve typical for a globular particle. The x-ray structure of IgFLNa20**–**21 (taken from the structure of IgFLNa19**–**21, PDB ID: 2J3S [23]) fits moderately to the scattering curve of IgFLNa20–21 (χ^2^ = 2.2, S2 Fig). Even though the published structure of IgFLNa20–21 lacks some loops, the addition of these loops does not change the fit to the SAXS data. The *D*_*max*_ (6.4 nm) measured from the x-ray structure is well in accordance with that obtained from the SAXS data (6.8 nm). In Fig 2A, the crystal structure of IgFLNa20**–**21 is overlaid with the averaged *ab initio* envelope of IgFLNa20**–**21 calculated from the SAXS data.

Migfilin binding led to notable changes in IgFLNa20**–**21 structural parameters. Both the Guinier plots and the distance distribution function *P(r)* showed that both *R*_*g*_ and *D*_*max*_ are increased upon peptide binding (Fig 2B and 2C, Table 1). Also, the shape of *P(r)* of IgFLNa20**–**21 is shifted from the typical bell-shaped curve of a globular particle into a more extended curve, suggesting a conformational change.

Based on earlier structural studies [25,51], it is apparent that part of the ligand-induced conformation changes of IgFLNa20**–**21 may be caused by the displacement of the first β strand of IgFLNa20 by the ligand. To study these and other changes caused by peptide ligands, we also analyzed a two-domain fragment lacking the first β strand, IgFLNaΔA20**–**21. SAXS measurements showed that IgFLNa∆A20**–**21 was significantly more elongated than IgFLNa20**–**21 (Table 1 and Fig 2). The shape of *P(r)* of IgFLNaΔA20**–**21 in the absence of peptide was similar to that of IgFLNa20**–**21 in the presence of peptide. Peptide addition did not significantly alter the *P(r)* of IgFLNaΔA20**–**21 (Fig 2C). The slightly bi-modal shapes of the *P(r)* curves suggest that in IgFLNa20**–**21 with the peptide and in IgFLNaΔA20**–**21 with or without the peptide, the two domains may be separated.

### Migfilin binding increases the conformational flexibility of IgFLNa20–21

The lack of domain-domain interactions might be apparent as flexibility of the particles in SAXS. To study this, the SAXS data was analyzed using dimensionless (normalized with *R*_*g*_ or volume-of-correlation *V*_*c*_) Kratky and Porod–Debye plots. In the dimensionless Kratky plot, the increase in flexibility is seen as a skewing of the parabolic shape of the curve [35,36]. The plots suggests that either migfilin binding or removal of the A strand increases the flexibility of the domain pair (Fig 2D; S3 Fig). In the presence of the peptide, both constructs show almost identical profiles, whereas without the peptide, IgFLNa20**–**21 is significantly more compact than IgFLNaΔA20**–**21 (Fig 2D). Similar behavior of these two constructs was observed also in the Porod–Debye plot, where the loss of a Porod plateau at low *q* angles is an indication of structural flexibility [36]. A clear plateau can be observed in the Porod–Debye plot of IgFLNa20**–**21 in the absence of peptide (Fig 2E), whereas with the bound peptide, no plateau can be seen, indicating increased flexibility upon peptide binding. For IgFLNaΔA20**–**21, no plateau is seen in the absence or presence of the peptide (Fig 2E).

### Solution-state modelling of peptide-bound IgFLNa20–21

**Fig 3:**
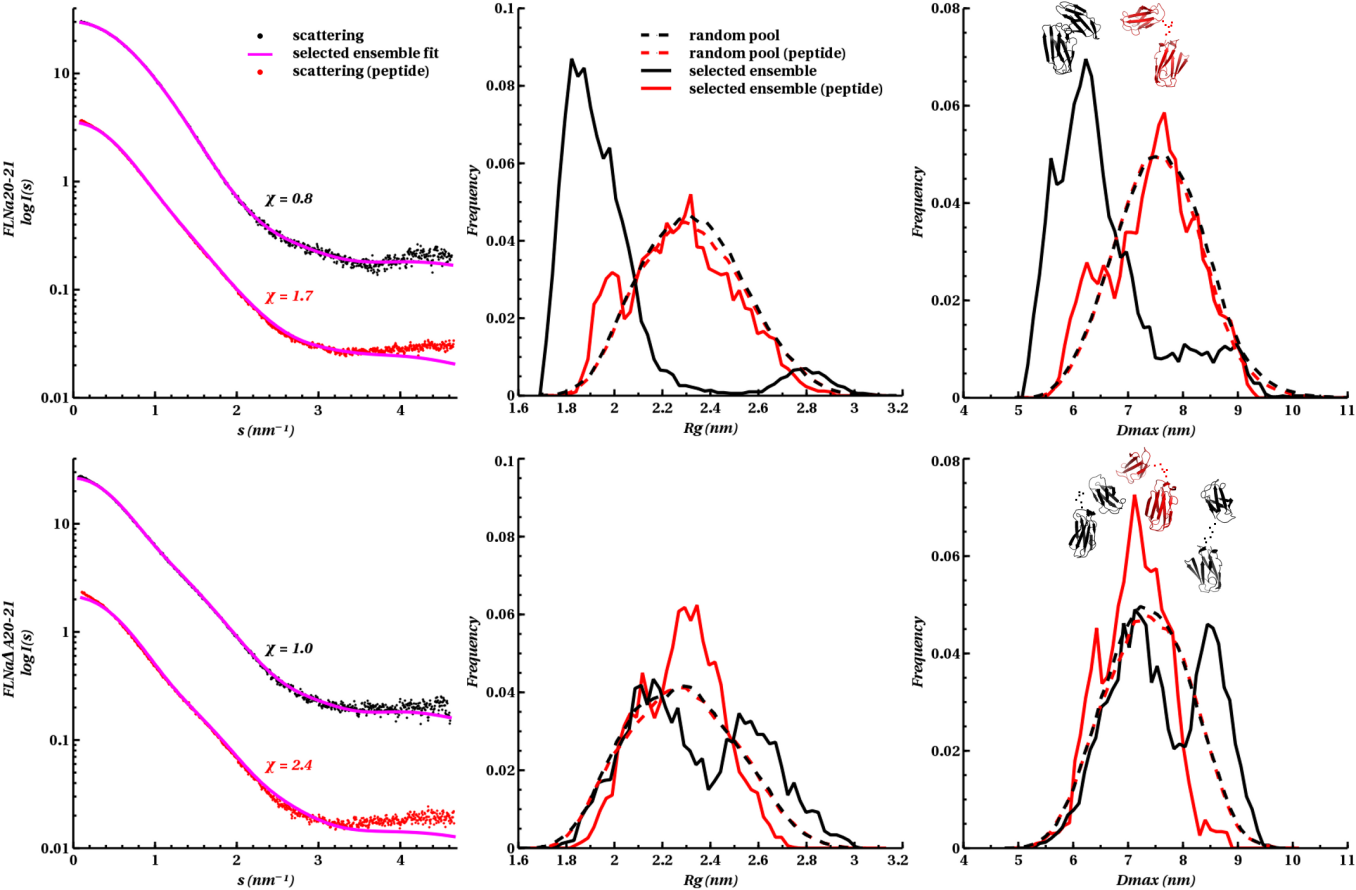
EOM modelling of migfilin peptide binding on IgFLNa(∆A)20–21. *Left panels*, Fit from the selected ensemble of conformers to the experimental scattering. Radius of gyration, *R*_*g*_ (*middle panels*), and particle maximum dimension, *D*_*max*_ (*right panels*), distribution histograms of the selected conformers versus the pool. Also shown in the right panel are examples of rigid body models of the selected conformers corresponding to the histogram peaks.

Kratky and Porod–Debye plots showed that the peptide-bound IgFLNa20**–**21 and IgFLNaΔA20**–**21 with or without peptide are flexible. Thus, traditional *ab initio* and rigid-body modeling techniques are not suitable for such particles with multiple conformations. Therefore, EOM analysis of the SAXS data was used to further model the conformational space of the two-domain fragments with and without peptide (Fig 3). Based on the EOM analysis, IgFLNa20**–**21 is mainly in a compact conformation with average *R*_*g*_ and *D*_*max*_ of 2.0 nm and 6.6 nm, respectively (Fig 3). *R*_*g*_ and *D*_*max*_ values are very similar to those obtained from the Guinier plots and *P(r)* function, and a similar *D*_*max*_ value can also be measured from the crystal structure of IgFLNa20**–**21. Interestingly, EOM-selected conformations also included a minor population of extended conformations with peaks in *R*_*g*_ and *D*_*max*_ around 2.8 nm and 8.5 nm, respectively. This explains the moderate fit of the IgFLNa20**–**21 crystal structure to the scattering data, as the scattering computed from the structure only represents the compact conformation (see above). Migfilin peptide-binding to IgFLNa20**–**21 changed the shape of the size distribution of the EOM-selected population compared to the non-bound one. With bound peptide, the size distribution of selected conformations is wide, with an average *R*_*g*_ and *D*_*max*_ of 2.3 nm and 7.5 nm, respectively (Fig 3). Accordingly, peptide binding to IgFLNa20**–**21 opens the compact two-domain fragment, also making it simultaneously more flexible, as the size distribution covers a wider range than without peptide.

The ΔA strand construct behaved differently in the EOM analysis than did the IgFLNa20**–**21 fragment. Without bound peptide, the EOM-selected population of IgFLNa∆A20**–**21 with a bimodal size distribution had one peak around 2.1 nm (*R*_*g*_) and 7.0 nm (*D*_*max*_) and another around 2.6 nm (*R*_*g*_) and 8.5 nm (*D*_*max*_). This suggests that IgFLNa∆A20–21 adopts two conformations in solution. The EOM-produced models are shown in Fig 3. Interestingly, peptide-binding to IgFLNaΔA20–21 changes the size distribution of the EOM-selected conformations from bimodal to unimodal. The size distribution is wide and very similar to that of IgFLNa20**–**21, with bound peptide having peaks at 2.3 nm (*R*_*g*_) and 7.5 nm (*D*_*max*_). Thus, the EOM analysis of the SAXS data suggests that in the presence of bound peptide, both IgFLNa20**–**21 and IgFLNaΔA20**–**21 populate a similar and rather wide conformation space.

### Crystal structure of IgFLNaΔA20–21 in complex with migfilin peptide

**Fig 4:**
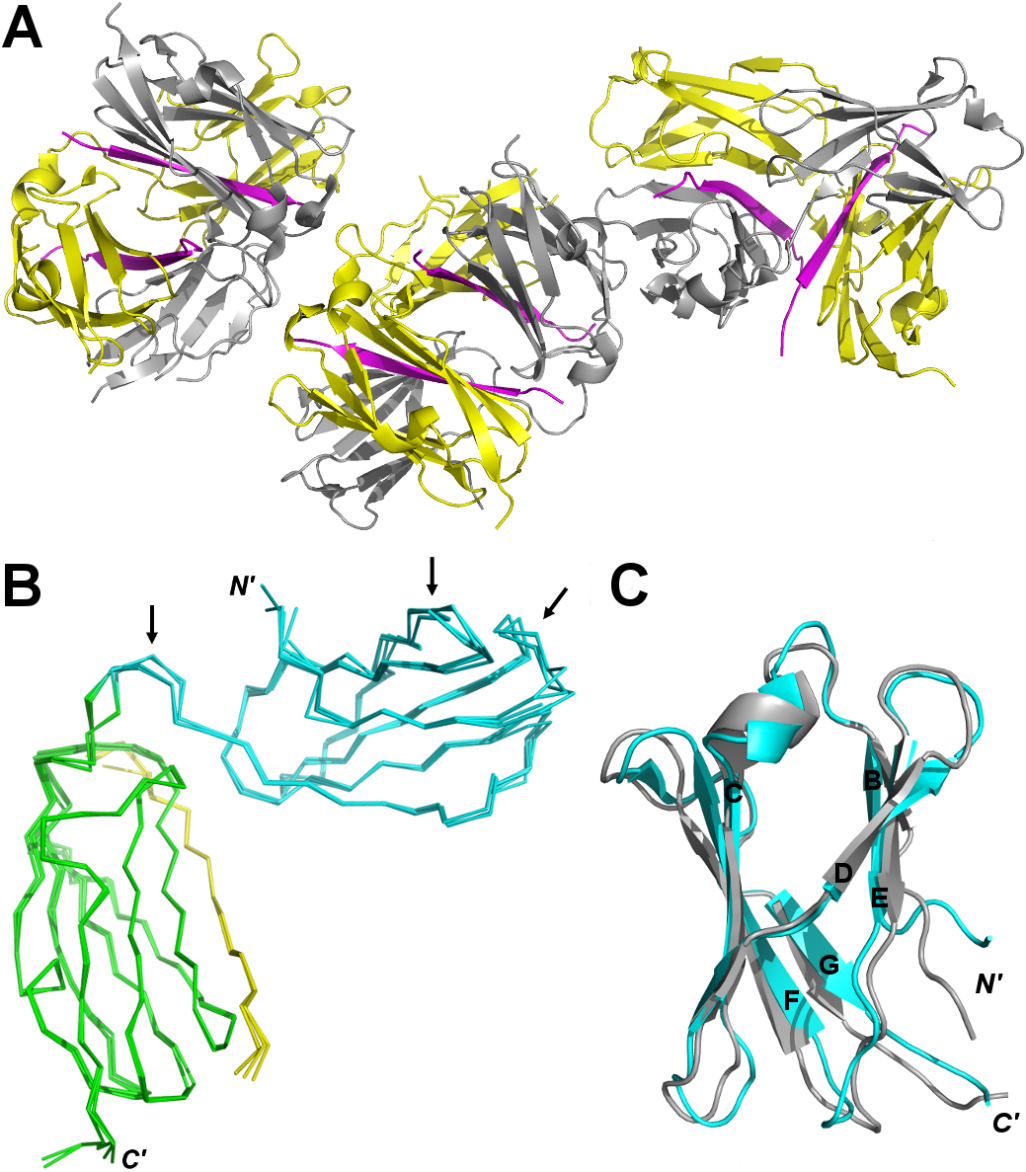
Crystal structure of IgFLNa∆A20–21-migflin peptide complex. **A** The asymmetric unit. FLN chains are colored in yellow and grey, and migfilin chains in magenta. **B** All six IgFLNa∆A20**–**21 chains of the asymmetric unit superimposed. All chains are nearly identical with each other. The greatest variation is seen in the inter-domain loop and at the loop regions of IgFLNa20 (shown with the black arrows). IgFLNa20 is colored in cyan, IgFLNa21 in green, and migfilin peptide in yellow. **C** Superimposition of IgFLNa20 (in cyan) from the crystal structure reported here and IgFLNb20 NMR structure (in gray) (2DLG). The structures are similar with CD faces adopting a closed conformation.

To obtain atomic detail information on structural changes caused by peptide-binding to the two domain fragments, we crystallized IgFLNaΔA20**–**21 in complex with the migfilin peptide. The A strand deletion construct was used to avoid unstructured sequences that might inhibit crystallization. The crystals belonged to the R3 space group, and diffraction data up to 2.0 Å resolution were used (Table 2). The asymmetric unit contained six copies of IgFLNaΔA20**–**21 assembled into three dimers (Fig 4A). In each dimer, the IgFLN polypeptide chains were crossed together by two migfilin peptides in between. In the final model, all six IgFLNaΔA20**–**21 molecules were nearly identical to each other (root-mean-square deviations of 0.0.6**–**0.39 Å for 139 Cα atoms). The greatest variation was seen at the loop regions of IgFLNa20 and at the inter-domain loop (Fig 4B). In chains A, B, C, and E, all loops in IgFLNa20 could be modeled, but in chains D and F, the electron density of loops between β strands of B**–**C and D**–**E was too poor to model these loops completely. The chains A, B, C, and E provide the first complete structure of domain 20 in isoform A, because in the earlier structure, many loops were missing as well [23]. The complete IgFLNa20 structure is similar to the previously solved NMR structure of the same domain in isoform B (PDB ID: 2DLG; root-mean-square deviation of 1.19 for 61 Cα atoms) (Fig 4C). The final *R*-factors of the refined IgFLNaΔA20**–**21 structure were *R*_*work*_ *=* 20.0 and *R*_*free*_ *=* 22.6. The structure fits only moderately with the experimental scattering of IgFLNa∆A20**–**21+migfilin (χ^2^ = 2.7, S2 Fig). This is in line with the flexibility analyses (Fig 2D and 2E) and EOM modelling (Fig 3), according to which the domain pair is flexible and adopts a rather wide range of different conformations.

**Table 2:** Data collection and refinement statistics for IgFLNa∆A20–21-migfilin complex structure.

Parameter	Statistic
Data collection	
Wavelength	0.93
Space group	R3
Cell dimensions	
a, b, c (Å)	88.15, 88.15, 394.71
α, β, γ (°)	90, 90, 120
Resolution range (Å)	43.86–2.00 (2.05–2.00)
Number of observations	
Total unique	76 965
Completeness (%)	99.6 (99.8)
Redundancy	3.65 (3.64)
*σ*	18.59 (2.18)
R_meas_ (%)	5.0 (58.2)
CC_0.5_ (%)	99.9 (83.3)
Refinement	
Resolution range (Å)	43.86–2.00
Number of reflections work/test set	73 110/3863
R_work_/R_free_	20.0/22.6
Deviation from ideal stereochemistry (RMSD)	
Bond lengths (Å)	0.02
Angles (°)	2.00
Ramachandran plot (%)	
Favored	98
Allowed	2
Outliers	0
Wilson B-factor (Å^2^)	40.6
PDB accession code	4P3W

The outer shell data are shown in parentheses.

**Fig 5:**
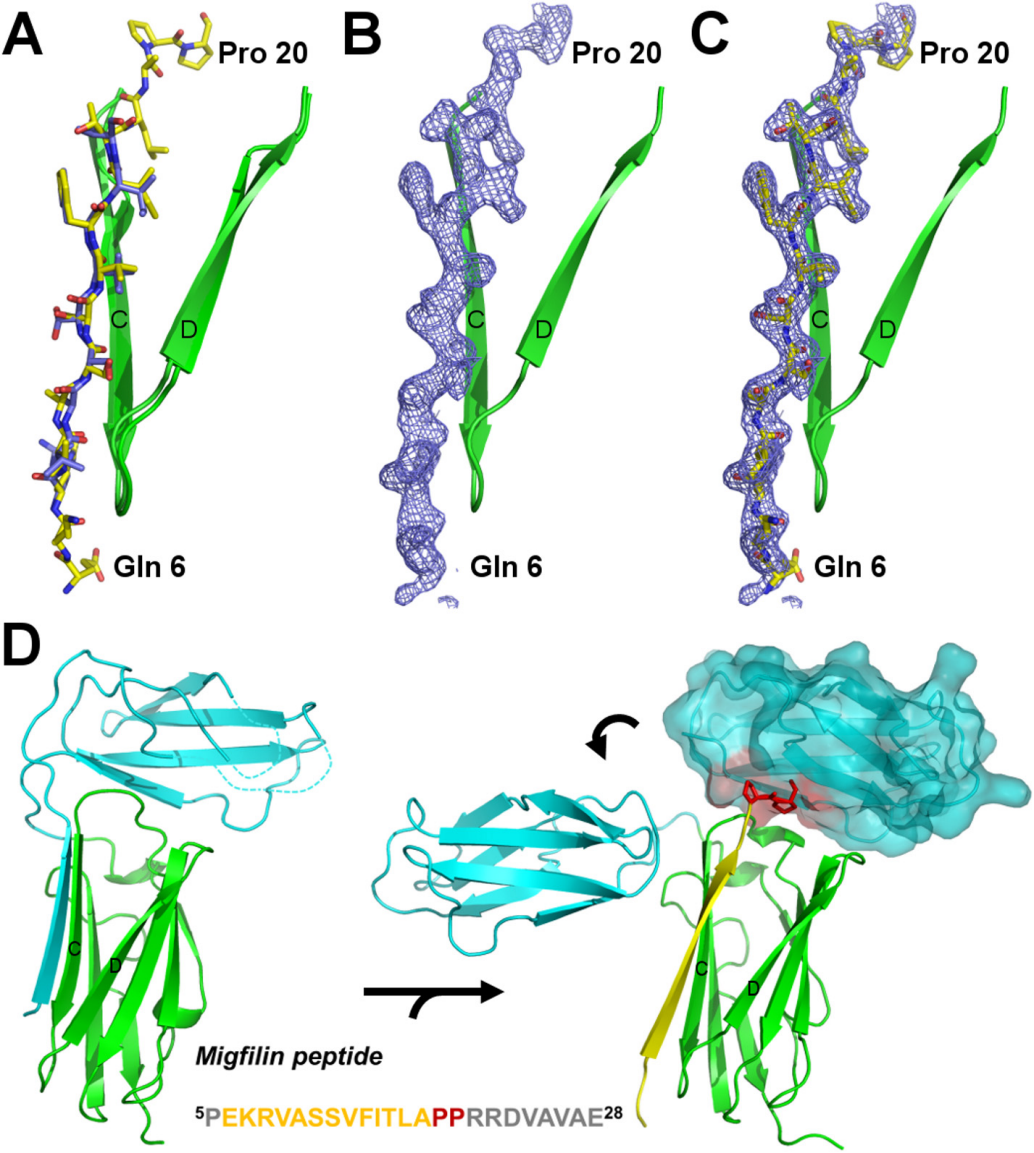
Migfilin peptide binding on IgFLNa∆A20–21. **A** Comparison of IgFLNa21-migfilin complex structure (peptide shown in orange) [18] (2W0P) with the structure reported here. Only the CD face and peptide are shown. **B** Electron density map (*F*_*o*_ *–F*_*c*_) of migfilin peptide shown at σ level 2.5 calculated from the refined model without the peptide. **C** Same as panel B with peptide shown. **D** Migfilin peptide binding causes major conformational changes in IgFLNa20**–**21. The left panel shows the compact structure of IgFLNa20**–**21 from 2J3S [23]. On the right panel, the current structure and the compact structure (2J3S) are superimposed for IgFLNa21 and the compact conformation of IgFLNa20 is shown surface rendered. The two proline residues in the migfilin peptide (shown in sticks) cause a steric clash (red) for the compact orientation of IgFLNa20. The peptide sequence is given below. Density for the amino acids in the peptide written in gray was not seen.

Migfilin residues 8–18 bind to the CD face of IgFLNa21 in the IgFLNaΔA20**–**21 construct, and prolines 19**–**20 bind to the top of domain 21, displacing domain 20 from the position seen in the crystal structure of FLNa19**–**21 [23] (Fig 5). These prolines do not form any interactions with IgFLNa21, but solely provide the necessary kink for the peptide to bind on top of the domain. Migfilin binding to the CD face is similar to that of the isolated domain 21 [18,52] (root-mean-square deviation of 0.36 Å for 89 atoms). Here we are also able to see migfilin residues 6**–**7, which were not seen in previous published structures [18,52]. However, these residues do not form any interactions with domain 21, although Lys^7^ together with Arg^8^ are thought to be important for the interaction with FLN [18,29].

### NMR analysis of IgFLNaΔA20–21

To obtain more information about the flexibility and interactions of IgFLNaΔA20**–**21, NMR measurements were performed without and with various concentrations of the migfilin peptide. Assignment was achieved with HNCACB and CBCA(CO)NH spectra. In addition, NMR diffusion data, ^15^N T1 and T2 relaxation data, as well as deuterium exchange data, were analyzed.

The NMR analysis of IgFLNaΔA20**–**21 in the presence of migfilin peptide suffered considerably from the disappearance of a large fraction of the IgFLNaΔA20 HSQC peaks upon peptide-binding. Most likely, this was due to severe line-broadening caused by an emerging exchange phenomenon in the intermediate, μs**–**ms timescale. No line-broadening is observed in the IgFLNa21 HSQC peaks, meaning that the observed peptide-induced exchange phenomenon is not caused by interdomain dynamics. Loss of the IgFLNaΔA20 peaks made it impossible to map changes at the interdomain interface. In spite of this, the NMR data could be used to evaluate the size of the protein, the binding site in IgFLNa21, and the domain-level flexibility of the protein.

The NMR diffusion data were used to deduce the radii of hydration for the free and peptide-bound IgFLNaΔA20**–**21. Radii of hydration derived from these data were 19.7±0.3 Å for the free form and 17.8±1.5 Å for the bound form. These values are in accordance with the values of 21.7 Å(free)/22.5 Å (bound) predicted from the number of residues [53] for a monomer. For the radius of hydration of a dimer, the prediction gives 26.4/27.4 Å.

**Fig 6:**
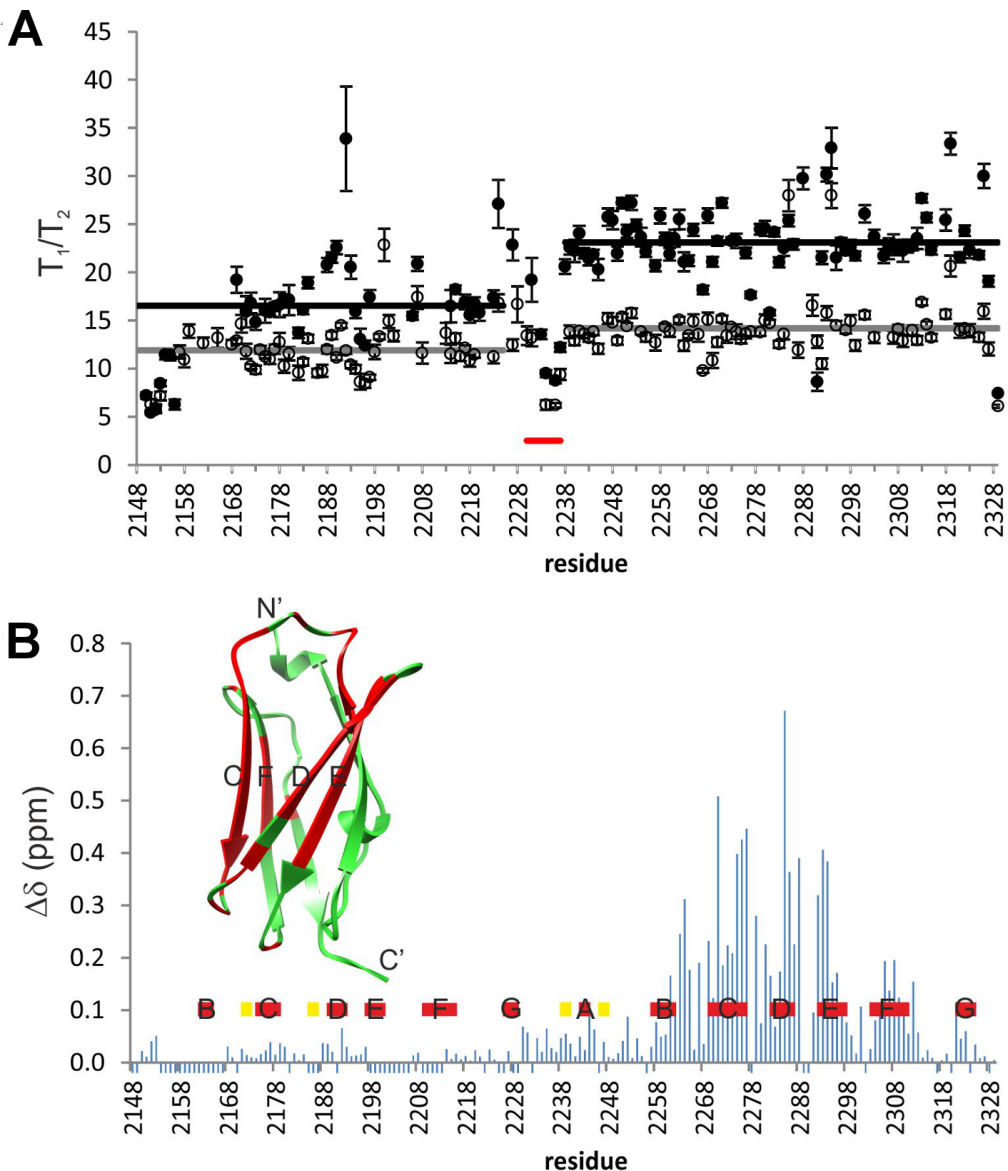
NMR spectroscopy of ^15^N-IgFLNa∆A20–21. **A** T_1_/T_2_ for free (open circles) and migfilin-bound (closed circles) ^15^N-IgFLNa∆A20**–**21. For each domain the average value is shown with a horizontal line. Domain linker region is indicated with a red bar. **B** Chemical shift perturbations in ^15^N-IgFLNa∆A20**–**21 upon titration with migfilin peptide. Residues corresponding to the β strands are shown with the red bars. Shift changes of ∆δ > 0.1 ppm are shown in the structure of IgFLNa21 in red. Residues with no data are assigned an arbitrary value -0.02 ppm.

To further measure the size and flexibility parameters of the domains, ^15^N T_1_ and T_2_ relaxation times were measured from free and peptide-bound IgFLNaΔA20**–**21 (S4 Fig). A plot of ^15^N T_1_/T_2_ versus the amino acid sequence is shown in Fig 6A. The average T_1_/T_2_ ratios are noticeably different for domains IgFLNaΔA20 and IgFLNa21, suggesting that the domains show no fixed relative orientation. In the absence of the peptide, the rotational correlation times of the individual domains, τ_c_, derived from the ^15^N T_1_/T_2_ ratios [54], were 9.8±0.2 ns for ΔA20 and 10.6±0.2 ns for 21. In the presence of peptide, the τ_c_ values were 11.9±0.2 ns for ΔA20 and 13.4±0.2 ns for 21. All of these values are significantly larger than those predicted based on the molecular weight of the individual domains (4.8 ns for ΔA20, 5.2 ns for 21, and 6.6 ns for 21+migfilin). The change of the domain-specific τ_c_ values upon peptide addition may be simply explained by the interaction of the peptide with IgFLNa21. The results suggest that the linkage between the two domains notably reduces the overall tumbling rates but allows for some interdomain flexibility. This is in accordance with the SAXS analysis presented above and has been observed and quantified for the wild-type domain pair when part of a larger assembly, IgFLNa16**–**21 [26].

To characterize the IgFLNaΔA20**–**21-migfilin interaction, we performed a peptide titration experiment and measured the peptide-induced chemical shift perturbations (Fig 6B, S5 Fig). In accordance with the crystal structure, significant chemical shift perturbations were located on the CD face of the IgFLNa21 domain and in the areas in immediate contact with the C and D strands. Upon titration, peaks of the free form disappear and those of the bound form appear as the peptide concentration is increased. Free and bound-form peaks are of approximately equal intensity in the HSQC spectrum acquired at a 1:1 protein-to-peptide ratio. These features are consistent with slow exchange in the NMR timescale, which is indicative of tight binding between IgFLNa21 and migfilin.

To gain more information about the structural dynamics in IgFLNaΔA20**–**21, H/D-exchange experiments were performed (S6 Fig). The initial data point in these experiments was acquired 18 min after solvent exchange. In the initial HSQC spectrum of the free form, there were 17 peaks from domain ΔA20. Only nine of these peaks were present in the initial spectrum of the bound form. This is in contrast to the situation in IgFLNa21. Here, the complex form of this domain was significantly more resistant to H/D exchange than that of the free form. The protected amide HSQC peaks of the complex form are clearly visible 63 h after the addition of D_2_O, whereas those of the free form vanish in less than 23 h. Increased protection is observed throughout the domain, not only at the immediate vicinity of the binding site. This suggests that the peptide brings additional stability to the whole IgFLNa21 domain.

Taken together, the NMR analysis supported the finding that IgFLNaΔA20**–**21 is a monomer in solution, both in the presence and the absence of the migfilin peptide. Furthermore, the relaxation analysis indicated that the two domains tumble partially independently, both in the absence and the presence of the peptide.

## Discussion

In this study, we have investigated the effect of ligand-binding on the conformation of the domain pair IgFLNa20–21. In this pair, IgFLNa21 is the highest-affinity binding site in FLN for many transmembrane protein cytoplasmic domains and for signaling adaptors such as migfilin [55]. This site is of considerable interest also because it has been shown to be regulated by mechanical forces that displace the A strand of IgFLNa20 in the pair, enabling interactions of IgFLNa21 [10,27].

Based on SAXS analysis, IgFLNa20**–**21 adopts mainly compact conformations, although a minor population of extended conformations is also predicted in EOM analysis. EOM-produced models suggest that the domains are separated in the extended conformations. The compact conformation of IgFLNa20**–**21 opens upon migfilin peptide-binding. Interestingly, the maximum dimensions of the peptide-bound IgFLNa20**–**21 interpose between the compact and extended forms seen without the bound peptide. Both the EOM analysis and the Kratky plot show that the ligand-bound IgFLNa20**–**21 is very flexible. The deletion of the A strand of domain 20 has a significant influence on the structure of this two-domain fragment. Both *ab initio* analysis of SAXS data and EOM modeling shows that the A strand deletion makes the IgFLNa20–21 fragment flexible. EOM analyses predicted a bimodal size distribution for IgFLNaΔA20**–**21, suggesting that it adopts two different extended conformation states, which are almost equally populated. The compact conformation seen with IgFLNa20**–**21 is not observed at all. EOM-produced models suggest that in both conformations, the domains are separated but the orientation of the domain-domain linker is altered. Accordingly, the A strand of domain 20 is needed for the rigidity of the mechanosensory domain pair 20**–**21. Interestingly, migfilin-binding to IgFLNaΔA20**–**21 reduces the conformational freedom of IgFLNaΔA20**–**21, as EOM produced a unimodal size distribution instead of bimodal, obtained without bound peptide. It is notable that the EOM-selected conformations for IgFLNaΔA20**–**21 with bound peptide are similar to those of IgFLNa20**–**21.

The crystal structure of IgFLNaΔA20**–**21 with migfilin peptide showed that migfilin binds to the CD face and binds to the top of IgFLNa21, displacing IgFLNa20 from the position seen in the crystal structure of IgFLNa19**–**21 [23]. In the crystal structure, the peptide also caused IgFLNaΔA20**–**21 to dimerize by being sandwiched between IgFLNa20 and IgFLNa21 of neighboring polypeptide chains. Both the SAXS and the NMR measurements revealed that in solution, intermolecular IgFLNaΔA20**–**21 complexes are not likely. Therefore, the observed interaction of IgFLNa20 and migfilin is merely a crystallization artefact.

**Fig 7:**
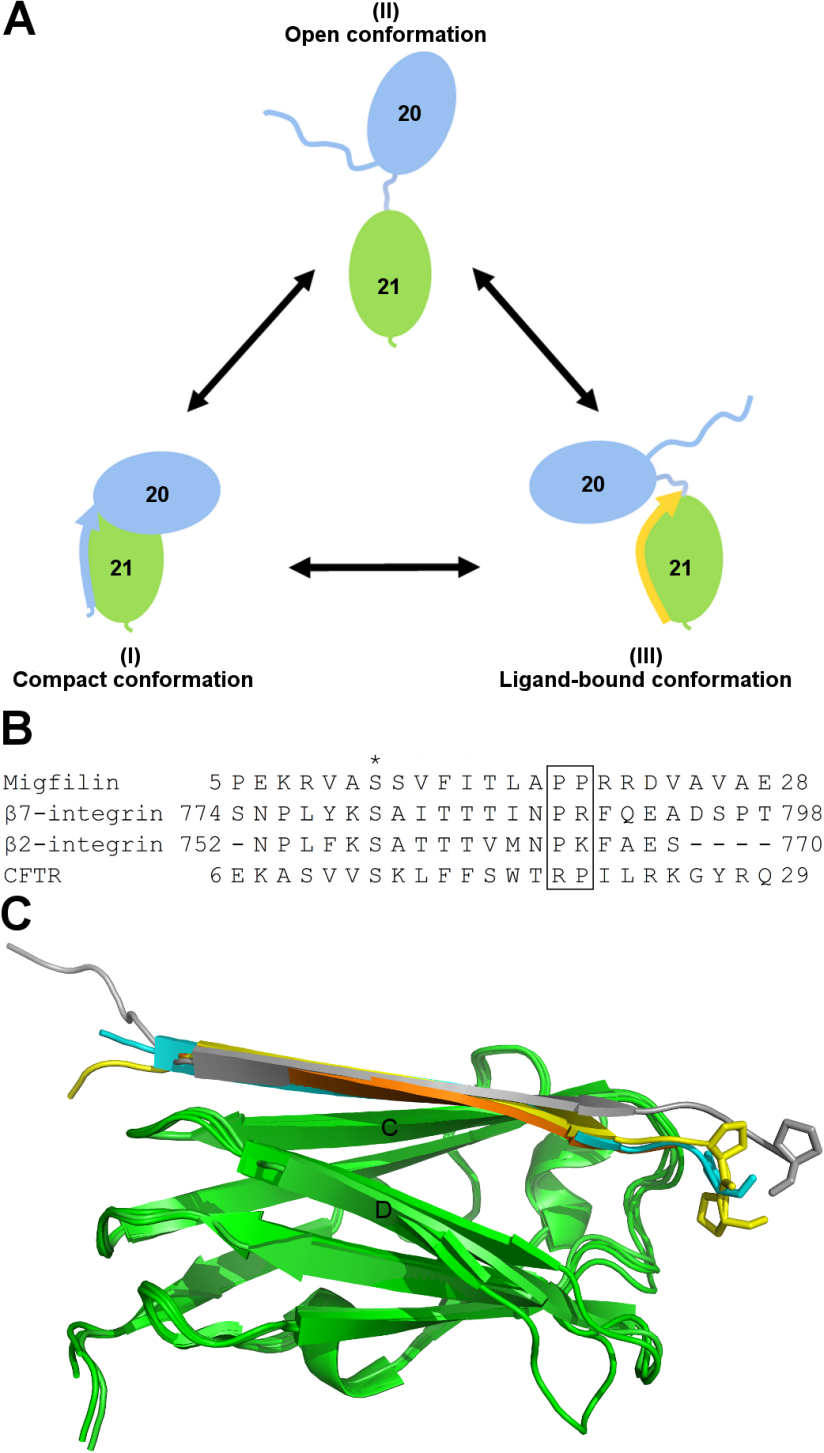
Peptide binding on IgFLNa20–21. **A** A model of three conformational states for the IgFLNa20**–**21 mechanosensor module. The compact conformation (I) domain pair interactions are disrupted by the replacement of the A strand. In open conformations (II) the domains move freely in relation to each other, but peptide ligand binding (III) restricts the movements. **B** Sequence alignment of the peptides known to interact with IgFLNa21 and whose IgFLN complex structures have been determined. **C** Superimposition of IgFLNa21 (green) structures in complex with peptides: migfilin (yellow), β7-integrin (cyan) [16], β2-integrin (orange) [19], and CFTR (grey) [20]. Prolines (shown as sticks) at C termini bend the peptides towards the top of IgFLNa21. The conformation of the CFTR peptide is influenced by crystal packing [20].

Combining our results with those reported earlier [8,27,28], we propose a model of three conformational states for the IgFLNa20**–**21 mechanosensor module shown in Fig 7A. The three states are: compact (I), open (II), and ligand-bound (III). SAXS-based EOM analysis shows that the majority of IgFLNa20**–**21 adopts a compact conformation (I). The compact conformation is also seen in the crystal structure of IgFLNa19**–**21 [23]. In the open state (II), IgFLNa20 and IgFLNa21 are detached from each other via the flexible domain-domain linker and form extended structures. In the open form, the domains can fluctuate rather freely in respect to each other. The open state is most prominent in the IgFLNaΔA20**–**21 construct when analyzed without peptide. Similar open conformations have previously also been obtained by applying external force on FLN [8,27]. Ligands bind either to the open (II) or the compact form (I), leading to conformations (III) that, based on EOM analysis, are less extended than the open forms (II). In the crystal structure of IgFLNaΔA20**–**21 with bound migfilin reported here, the migfilin peptide binds on top of IgFLNa21. Accordingly, the peptide-binding to the top of domain 21 might restrict the mobility of the linker peptide between IgFLNa20 and IgFLNa21 (Fig 5D), thus preventing FLN to adopt the open conformations (II). It should be noted that in the crystal structure, only the migfilin residues 6**–**20 of the total 5**–**28 used in the crystallization and SAXS experiments are seen. Accordingly, there are eight C-terminal amino acids whose locations are not known based on the crystal structure.

The three conformation states of I, II, and III described above can be predicted to be in equilibrium exchange (Fig 7A). Although IgFLNa20**–**21 mainly adopts the compact conformation I, EOM analysis suggests that a small population of IgFLNa20**–**21 can also adopt the open conformations (II). This is in accordance with recent single-molecule force spectroscopy measurements in which small pN-range external forces shifted the equilibrium between open and closed states of IgFLNa20**–**21 towards the open state, but extrapolation to zero force predicted that occasional opening can take place without force [27]. Peptide-binding to the open conformation is reversible and the dissociation constants are similar to those for individual IgFLNa21, ranging between 150 nM and 500 μM [16,18,19,23,55]. Recent FRET studies *in vitro* and in cultured cells have shown that IgFLNa20**–**21 domain separation can be induced by ligand peptides as well as by mechanical force [28]. It would be interesting if the FRET probes could be refined so that the differences in domain distances and the orientations in compact and open conformations could be monitored.

Do other IgFLNa21-binding partners cause ligand-bound conformations of the IgFLNa20**–**21 domain pair similar to what we see here with the migfilin peptide? The complex structures of IgFLNa21 with four different binding partners has been solved [16,18–20]. All of these partners bind similarly to the CD face of IgFLNa21, and the sequence alignment shows at least one proline and a bulky and charged amino acid immediately following the β-strand-forming residues (Fig 7B and 7C). The conserved prolines are predicted to bring structural rigidity to the peptide and thus the alignments and structures suggest that all peptides could restrict movement of IgFLNa20, leading to a similar ligand-bound state as reported here for migfilin. Interestingly, IgFLNa18**–**19 also adopts an auto-inhibited compact conformation similar to that of IgFLNa20**–**21 [24], and IgFLNa19 serves as a binding site for many of the same proteins as IgFLNa21, albeit with a lower affinity [55]. It is possible that the three-conformational-states model presented here also holds for IgFLNa18**–**19.

In conclusion, both the solution-state modelling and the crystal structure suggest that the ligand-bound conformations of the IgFLNa20**–**21 domain pair are unique and differ from the compact or open conformations. This may be an important feature in the mechanosensor function of the site.

## Supporting Information

S1 Fig Analysis of the concentration dependence of the SAXS data. Raw experimental x-ray scattering data for each step (1, 2.5, and 4 mg/ml) of the measured concentration series for IgFLNa20**–**21 and IgFLNa∆A20**–**21 with and without peptide. Left panel: the experimental scattering shown scaled to the same forward scattering intensity *I(0)*. Middle panel: Guinier analyses arbitrarily placed on the y axis. Right panel: Normalized distance distribution function *P(r)*.
(TIFF)

S2 Fig Crystal structure fits to the experimental scattering. Fit of **A** IgFLNa20**–**21 (from IgFLNa19–21 crystal structure, PDB ID: 2J3S [23]) and **B** IgFLNaΔA20–21+miglifin complex structure (current structure) to the respective experimental solution scattering profile.
(TIFF)

S3 Fig Volume-of-correlation *V*_*c*_ normalized Kratky plot. (TIFF)

S4 Fig Longitudinal ^15^N R_1_ and transverse ^15^N R_2_ relaxation rates for free and migfilin-bound IgFLNa∆A20–21. (TIFF)

S5 Fig HSQC spectra of NMR measurements. Overlaid HSQC spectra of free IgFLNa∆A20**–**21 (red, green for aliased peaks) and IgFLNa∆A20**–**21 after addition of migfilin peptide at a ratio of 5:1 peptide to IgFLNa∆A20**–**21 (blue, cyan). Peaks with ∆δ > 0.2 ppm are indicated with residue numbers.
(TIFF)

S6 Fig H/D exchange experiments performed for free and migfilin-bound IgFLNa∆A20–21. **A** Overlay of HSQC spectra of free IgFLNa∆A20–21 in 95/5% H_2_O/D_2_O (red contour) and in 100% D_2_O (green contours) after a 18 min sample preparation time. **B** As in **A,** but for the complex form. Peaks in the exchanged spectra are assigned with residue numbers. Residues 2151–2235 form domain ∆A20 and 2236–2329 domain 21. Peaks without assignment have overlapping or no assignments in the reference spectra. **C–D** Exchange protected residues mapped on the structures of the free (PDB ID: 2J3S) and bound form, respectively. Strand A in the free form and migfilin peptide in the bound form are shown in yellow.
(TIFF)

S1 Table SAXS data collection parameters and data analysis software. (DOCX)

S2 Table SAXS-derived sample parameters. (DOCX)
