# Supplementary figures and images for: Flexible Structure of Peptide-Bound Filamin A Mechanosensor Domain Pair 20–21
Source: PLoS One. 2015 Aug 31;10(8):e0136969. doi: 10.1371/journal.pone.0136969 (PMC4554727; doi:10.1371/journal.pone.0136969)

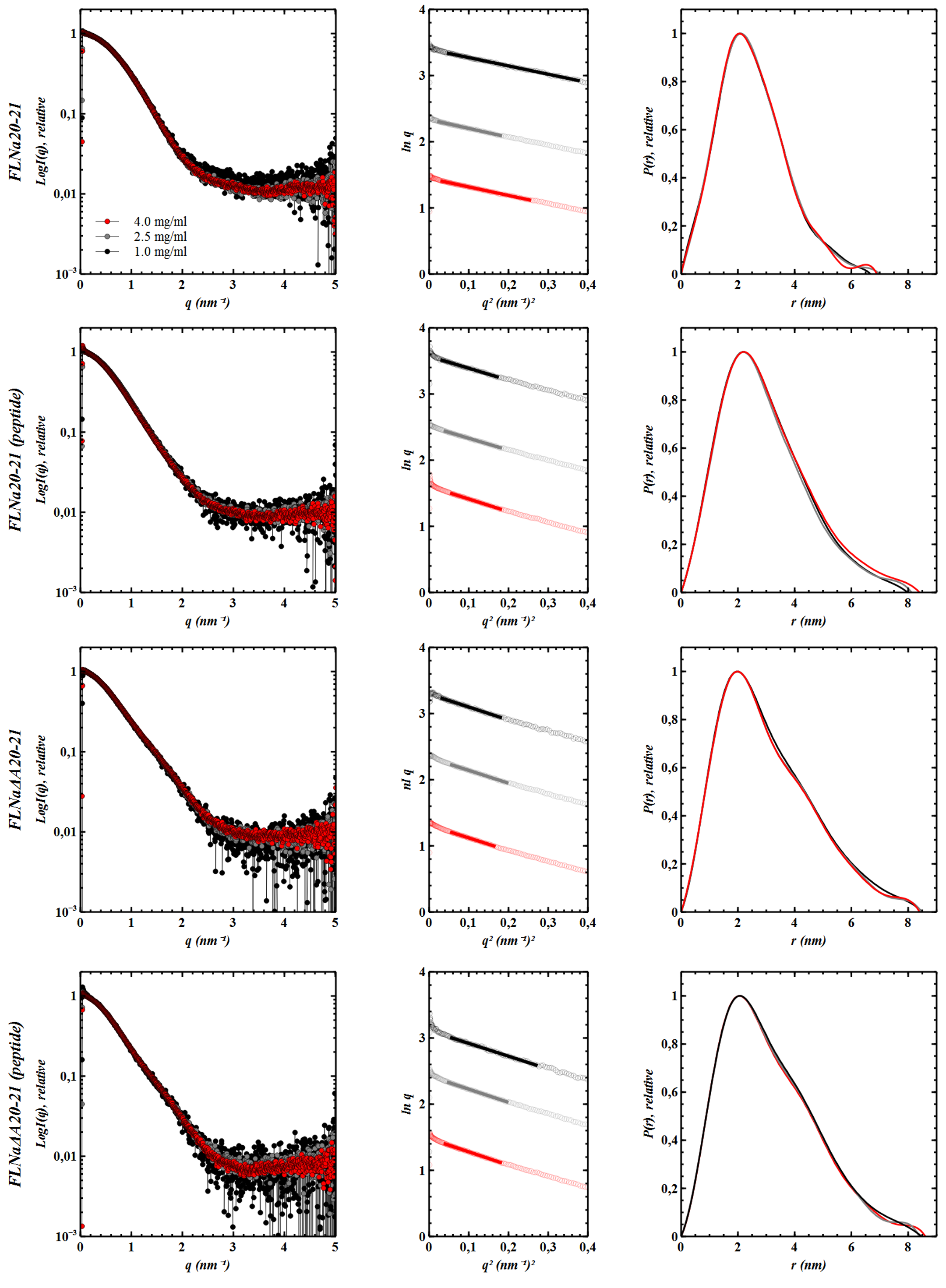

Supplement: S1 Fig — Raw experimental x-ray scattering data for each step (1, 2.5, and 4 mg/ml) of the measured concentration series for IgFLNa20–21 and IgFLNa∆A20–21 with and without peptide. Left panel: the experimental scattering shown scaled to the same forward scattering intensity I(0). Middle panel: Guinier analyses arbitrarily placed on the y axis. Right panel: Normalized distance distribution function P(r). (TIFF) [file pone.0136969.s001.tiff]

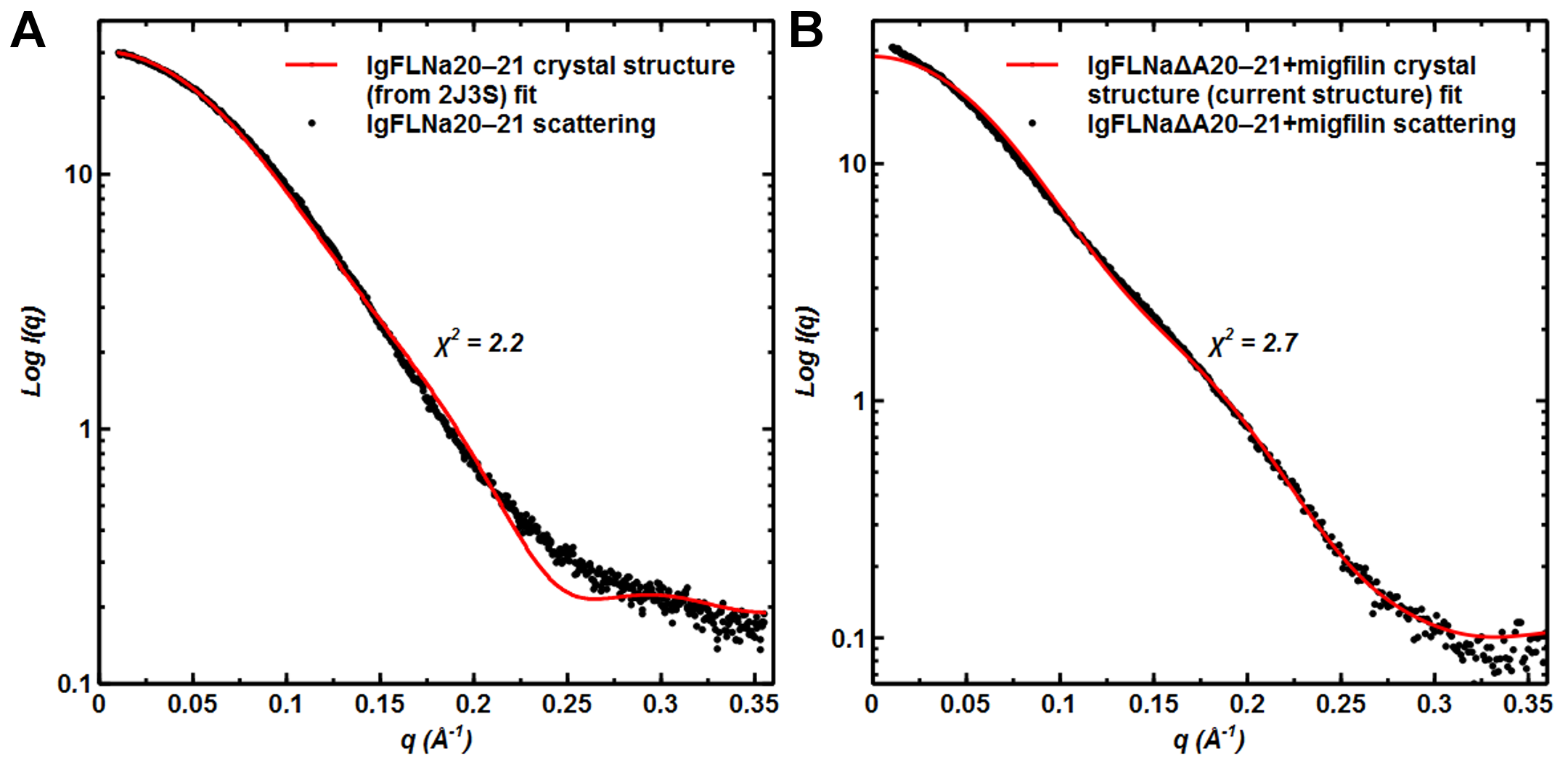

Supplement: S2 Fig — Fit of A IgFLNa20–21 (from IgFLNa19–21 crystal structure, PDB ID: 2J3S [23]) and B IgFLNaΔA20–21+miglifin complex structure (current structure) to the respective experimental solution scattering profile. (TIFF) [file pone.0136969.s002.tiff]

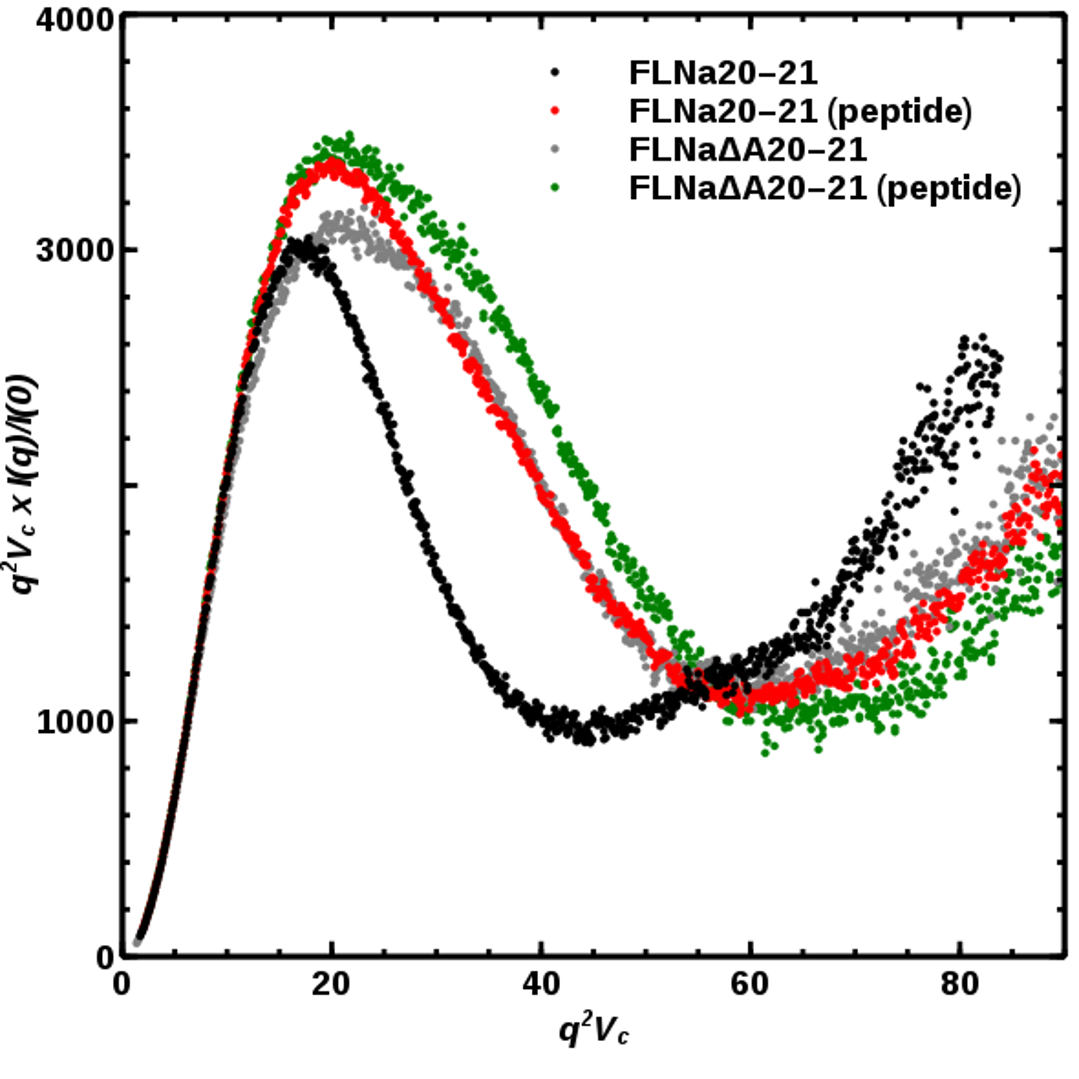

Supplement: S3 Fig — (TIFF) [file pone.0136969.s003.tiff]

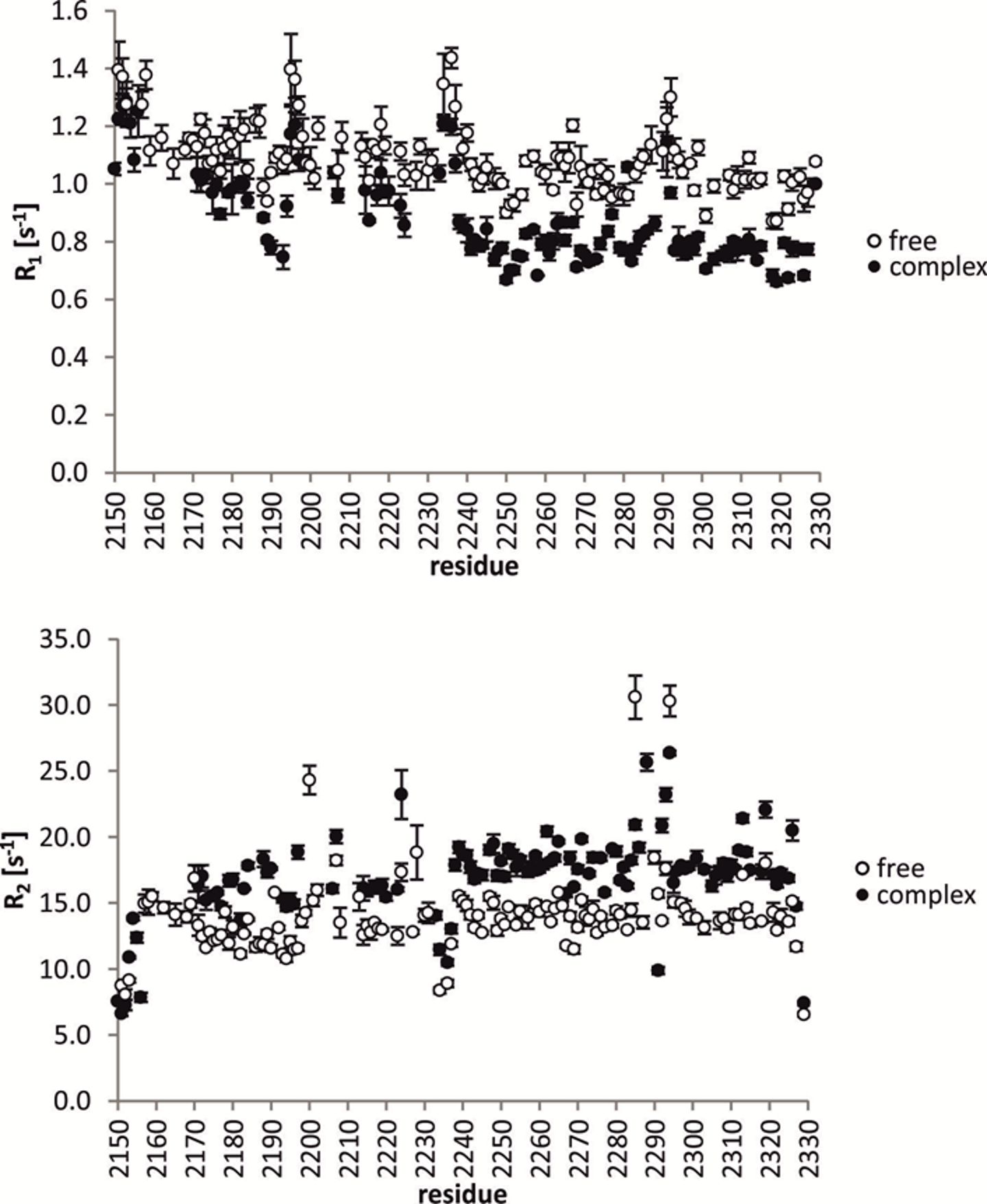

Supplement: S4 Fig — (TIFF) [file pone.0136969.s004.tiff]

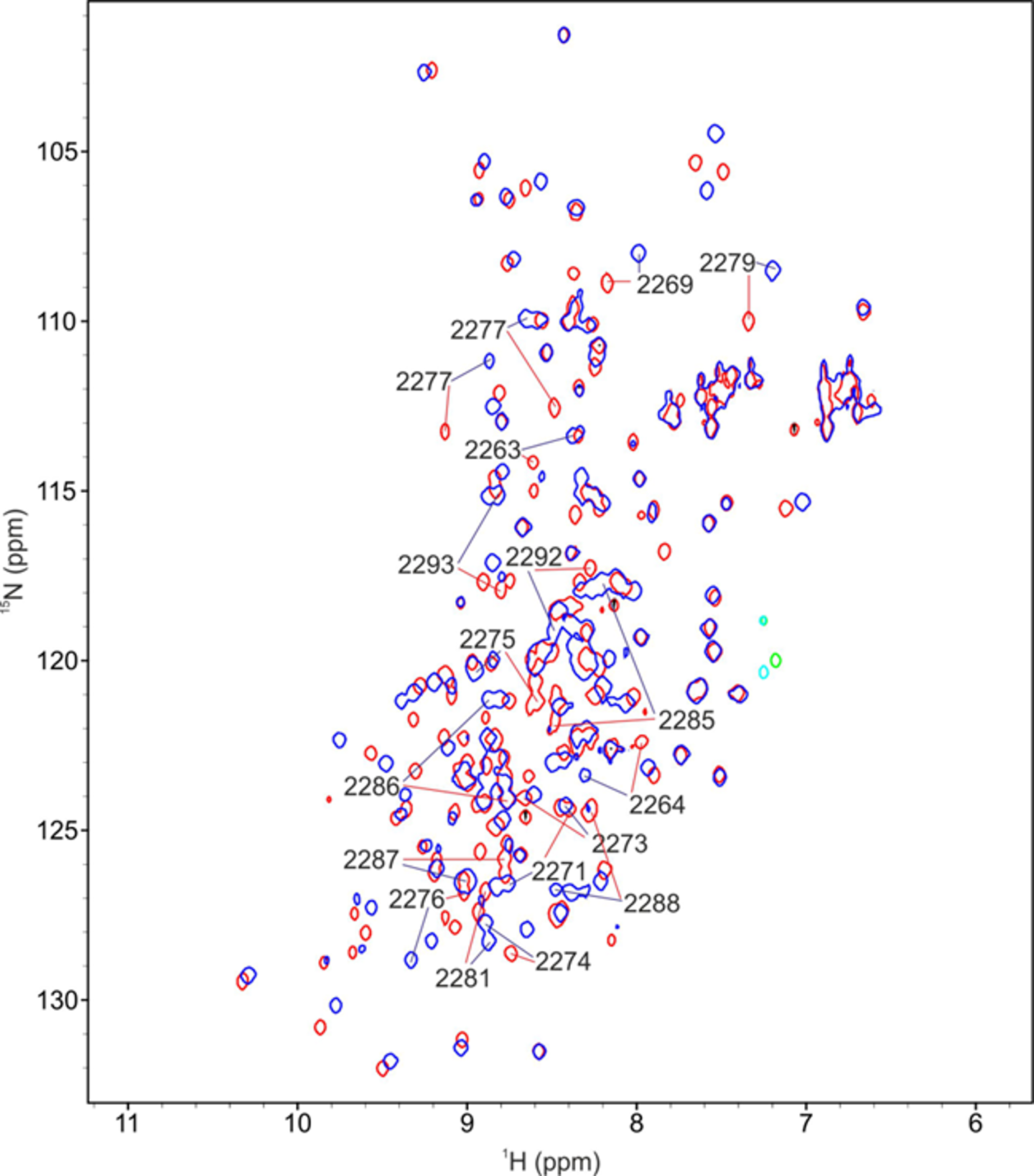

Supplement: S5 Fig — Overlaid HSQC spectra of free IgFLNa∆A20–21 (red, green for aliased peaks) and IgFLNa∆A20–21 after addition of migfilin peptide at a ratio of 5:1 peptide to IgFLNa∆A20–21 (blue, cyan). Peaks with ∆δ > 0.2 ppm are indicated with residue numbers. (TIFF) [file pone.0136969.s005.tiff]

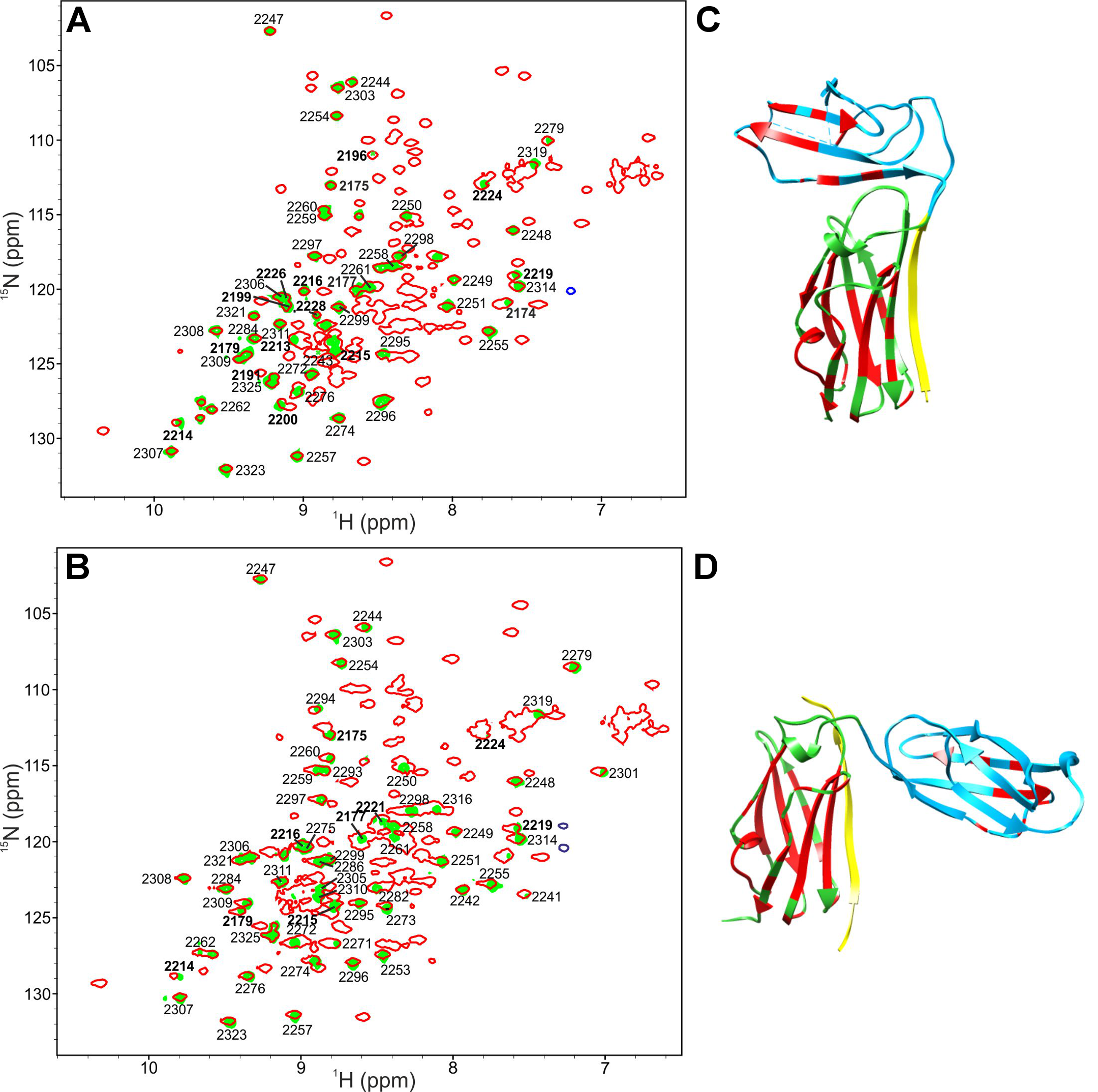

Supplement: S6 Fig — A Overlay of HSQC spectra of free IgFLNa∆A20–21 in 95/5% H2O/D2O (red contour) and in 100% D2O (green contours) after a 18 min sample preparation time. B As in A, but for the complex form. Peaks in the exchanged spectra are assigned with residue numbers. Residues 2151–2235 form domain ∆A20 and 2236–2329 domain 21. Peaks without assignment have overlapping or no assignments in the reference spectra. C–D Exchange protected residues mapped on the structures of the free (PDB ID: 2J3S) and bound form, respectively. Strand A in the free form and migfilin peptide in the bound form are shown in yellow. (TIFF) [file pone.0136969.s006.tiff]
