# Supplementary material for: Flexible Structure of Peptide-Bound Filamin A Mechanosensor Domain Pair 20–21
Source: PLoS One. 2015 Aug 31;10(8):e0136969. doi: 10.1371/journal.pone.0136969 (PMC4554727; doi:10.1371/journal.pone.0136969)
Supplement: S1 Table — (DOCX) [file pone.0136969.s007.docx]

**S1 Table. SAXS data collection parameters and data analysis software.**

| **Data collection parameters** |  |
| --- | --- |
| Beamline | ESRF BM**–**29 |
| Beam geometry, mm | 0.70 by 0.70 |
| Wavelength, nm | 0.10 |
| *q* range, nm^-1^ | 0.01–5.0 |
| sample-detector distance, m | 2.85 |
| Exposure time, sec | 1.0 |
| Concentration range, mg ml^-1^ | 1.0–4.0 |
| Temperature, K | 277 |
| **Software employed** |  |
| Primary data reduction | BsxCuBE |
| Data processing | PRIMUS |
| *Ab initio* analysis | Gasbor |
| Validation and averaging | Damaver |
| Rigid-body modeling | EOM |
| Computation of model intensities | Crysol |
| Superimposition of models | Supcomb |
| 3D graphics representations | PyMOL |
