# Supplementary material for: Flexible Structure of Peptide-Bound Filamin A Mechanosensor Domain Pair 20–21
Source: PLoS One. 2015 Aug 31;10(8):e0136969. doi: 10.1371/journal.pone.0136969 (PMC4554727; doi:10.1371/journal.pone.0136969)
Supplement: S2 Table — (DOCX) [file pone.0136969.s008.docx]

**S2 Table. SAXS-derived sample parameters.**

|  | **IgFLNa20–21** | **IgFLNa20–21 +migfilin** | **IgFLNa∆A20–21** | **IgFLNa∆A20–21 +migfilin** |
| --- | --- | --- | --- | --- |
| **Structure parameters** |  | | | |
| *I*(0), cm^-1^ [from *P(r)*] | 30.0 | 30.1 | 29.9 | 30.1 |
| *R_g_*, nm [from *P(r)*] | 2.0 | 2.4 | 2.4 | 2.5 |
| *I*(0), cm^-1^ [from Guinier] | 30.0 | 30.0 | 30.0 | 30.1 |
| *R_g_*, nm [from Guinier] | 1.9 | 2.3 | 2.4 | 2.4 |
| *D_max_*, nm | 6.8 | 8.2 | 8.5 | 8.2 |
| Porod volume estimate, nm^3^ | 32.0 | 32.9 | 28.9 | 31.1 |
| Dry volume calculated from sequence, nm^3^ | 24.1 | 26.0 | 22.5 | 25.3 |
| **Molecular mass determination** |  | | | |
| Molecular mass *M_r_* [*I(0)*], kDa ^a^ | 30.0 | 30.0 | 30.0 | 30.0 |
| Calculated monomeric *M_r_* from sequence, kDa | 20.0 | 22.6 | 18.7 | 21.3 |

The values are for merged scattering of high and low concentration data.

^a^ Sample forward scattering intensity *I(0)* was compared to that of reference solution of bovine serum albumin (*M_r_* 66.5 kDa) using the following equation: *M_w (sample)_* = *I(0)_sample_* x *M_w (BSA)_* / *I(0)_BSA_* .
